# In-Vivo Optical Monitoring of the Efficacy of Epidermal Growth Factor Receptor Targeted Photodynamic Therapy: The Effect of Fluence Rate

**DOI:** 10.3390/cancers12010190

**Published:** 2020-01-13

**Authors:** Wei Peng, Henriette S. de Bruijn, Timo L. M. ten Hagen, Kristian Berg, Jan L. N. Roodenburg, Go M. van Dam, Max J. H. Witjes, Dominic J. Robinson

**Affiliations:** 1 ErasmusMC Cancer Institute, Department of Otolaryngology and Head & Neck Surgery, Center for Optical Diagnostics and Therapy, Dr. Molenwaterplein 40, 3015 GD Rotterdam, The Netherlands; 2 Department of Oral and Maxillofacial Surgery, University Medical Center Groningen, Hanzeplein 1, 9713 GZ Groningen, The Netherlands; 3 ErasmusMC, Laboratory of Experimental Oncology, Department of Pathology, Dr. Molenwaterplein 40, 3015 GD Rotterdam, The Netherlands; 4 Department of Radiation Biology, Institute for Cancer Research, Norwegian Radium Hospital, Oslo University Hospital, Boks 1072 Blindern, NO-0316 Oslo, Norway; 5 Department of Pharmacy, School of Pharmacy, University of Oslo, Boks 1072 Blindern, NO-0316 Oslo, Norway; 6 Department of Surgery, Nuclear Medicine and Molecular Imaging and Intensive Care, University of Groningen, University Medical Center Groningen, Hanzeplein 1, 9713 GZ Groningen, The Netherlands

**Keywords:** targeted, EGFR, photosensitizer, photodynamic therapy, intravital microscopy, growth delay

## Abstract

Targeted photodynamic therapy (PDT) has the potential to improve the therapeutic effect of PDT due to significantly better tumor responses and less normal tissue damage. Here we investigated if the efficacy of epidermal growth factor receptor (EGFR) targeted PDT using cetuximab-IRDye700DX is fluence rate dependent. Cell survival after treatment with different fluence rates was investigated in three cell lines. Singlet oxygen formation was investigated using the singlet oxygen quencher sodium azide and singlet oxygen sensor green (SOSG). The long-term response (to 90 days) of solid OSC-19-luc2-cGFP tumors in mice was determined after illumination with 20, 50, or 150 mW·cm^−2^. Reflectance and fluorescence spectroscopy were used to monitor therapy. Singlet oxygen was formed during illumination as shown by the increase in SOSG fluorescence and the decreased response in the presence of sodium azide. Significantly more cell death and more cures were observed after reducing the fluence rate from 150 mW·cm^−2^ to 20 mW·cm^−2^ both in-vitro and in-vivo. Photobleaching of IRDye700DX increased with lower fluence rates and correlated with efficacy. The response in EGFR targeted PDT is strongly dependent on fluence rate used. The effectiveness of targeted PDT is, like PDT, dependent on the generation of singlet oxygen and thus the availability of intracellular oxygen.

## 1. Introduction

Photodynamic therapy (PDT) involves the administration of a photosensitizer (PS) that after exposure to light, and in the presence of oxygen, leads to the formation of reactive oxygen species, predominantly singlet oxygen [1]. The efficacy of the treatment is dependent on many factors that dynamically interact during treatment. Ground state oxygen is needed for formation of singlet oxygen, which means that tissue oxygenation strongly influences treatment efficacy [2]. Illumination with high fluence rates can deplete oxygen levels in both tumor and normal tissue, limiting efficacy [3,4]. Vascular responses that can occur during PDT, such as constriction and thrombus formation, can additionally decrease tumor oxygenation and negatively affect the PDT response [5,6,7,8]. Conversely, vascular responses may potentially enhance tumor necrosis due to vascular occlusion [5,9]. The dynamic interaction between light, photosensitizer, cells and vasculature can potentially change optical properties influencing light delivery to tissue. Therefore, a full understanding of light delivery, tissue optical properties, oxygenation levels and photosensitizer localization are important if PDT is to be optimized. We and others have shown that rate of fluorescence photobleaching of some photosensitizers during PDT is related to formation of singlet oxygen and can be used for implicit dosimetry [3,10,11,12]. Most fluorescence measurements techniques however do not account for the influence of tissue optical properties on fluorescence signals that have been shown to be different for different tissues and change during PDT. Single fiber reflectance (SFR) spectroscopy combined with fluorescence (SFF) spectroscopy can be used to determine the absorption and scattering properties of light through tissue and to calculate the intrinsic fluorescence intensity [13]. These types of in-situ measurements during treatment have potential for monitoring the efficacy of PDT.

Targeted strategies to deliver PSs specifically to tumor cells have the potential to improve the therapeutic effect of PDT due to significantly better tumor responses, less normal tissue damage and decreased skin photosensitization [1,14,15]. In this approach different, and in many cases increased, expression levels of receptors on tumor cells can be used as a target. In head and neck cancer it has been shown that 83% of tumors show overexpression of epidermal growth factor receptor (EGFR) which is commonly utilized in targeted therapies [16]. One way to target a PS to the EGFR receptor is to conjugate with an antibody, first reported by Mew et al. [17]. The phthalocyanine dye IRDye700DX, is a hydrophilic PS that by itself has little photodynamic efficacy due to a poor cellular localization and is commonly used to form a conjugate with a targeting molecule. Cetuximab (Erbitux^TM^) is an antibody against human EGFR that is approved by the FDA and EMA to treat colorectal and late stage head and neck cancer. A conjugate of cetuximab and IRDye700DX (cetuximab-IRDye700DX) has the potential for effective photosensitizer delivery to tumor cells and has been shown to cause selective tumor destruction after light irradiation [18,19,20,21,22,23,24]. In-vitro studies investigating cetuximab-IRDye700DX have shown cell membrane localization and rapid internalization, and cell death after light exposure [20,21,23]. Sato et al. [20] and Nagaya et al. [21] both demonstrated a delay in growth of a preclinical tumor after cetuximab-IRDye700DX PDT using either one light fluence or repetitive treatments. Recently we have shown in an intravital imaging study that tumor localization, minimal vascular responses and PDT induced tumor necrosis occur using an illumination scheme with a single fluence of 100 J·cm^−2^ delivered at a fluence rate of 50 mW·cm^−2^ [24]. To our knowledge the effect of different fluence rates on the efficacy of targeted PDT has not been investigated in-vivo.

Cetuximab-IRDye700DX is currently under investigation for the treatment of recurrent head and neck squamous cell carcinoma in a phase III trial [25]. In a phase I/IIb clinical trial of 30 patients, Gillenwater et al. [26] show promising responses using a drug-light-interval (DLI) of 24 h between drug administration and light exposure for 4 to 6 min. Although explicit details on the treatment parameters (fluence and fluence rate) were not reported, the short illumination times described means it is most likely that fluence rates were high, because this reduces illumination times, and in the range of 150 mW·cm^−2^. Only mild and moderate treatment related side effects such as tumor hemorrhage, swelling and pain were observed.

These promising preclinical and clinical results encourage us to investigate the effects of fluence rate on cetuximab-IRDye700DX mediated PDT. The use of a photosensitizer with a moderate singlet oxygen quantum yield suggests that photodynamic action is the source of damage in antibody targeted IRDye700DX mediated PDT. The literature on this subject is however contradictory, some studies performed experiments in the presence of reactive oxygen speciesor singlet oxygen scavengers and could not completely remove the effect leading authors to invoke other mechanisms of cell death [18,19] whereas other studies have shown a singlet oxygen only mechanism [22]. In designing strategies for treatment optimization it is important to know to what extent singlet oxygen mediates PDT response and if oxygen availability is a limiting factor.

Therefore in the present study we investigate the involvement of singlet oxygen and the effect of different fluence rates on head and neck cancer derived OSC-19-luc2-cGFP, scc-U2 and scc-U8 cells in-vitro. We continue this study in-vivo investigating long-term effects of different fluence rates on the growth of a subcutaneous OSC-19-luc2-cGFP solid tumor in the flank of a mouse. We hypothesize that treatment with a low fluence rate will be more effective due to efficient use of the tissue oxygenation. We utilize single fiber reflectance spectroscopy and fluorescence spectroscopy to monitor PDT response during illumination and investigate whether there was a correlation between this and treatment efficacy [27]. Our results show that the response to EGFR targeted PDT is the result of singlet oxygen formation, that this response is dependent on the fluence rate used, and that in-vivo optical monitoring of IRDye700DX fluorescence photobleaching has potential for predicting therapeutic efficacy.

## 2. Results

### 2.1. Effect of Different Fluence Rates In Vitro

**Figure 1:**
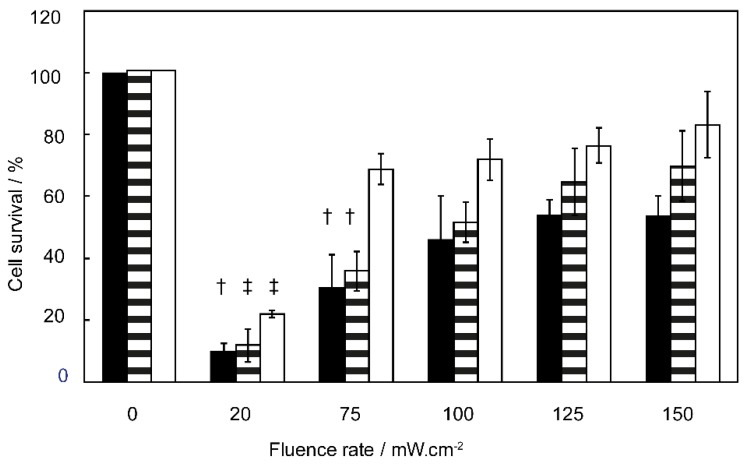
Cell survival of OSC-19-luc2-cGFP (black bar), scc-U2 (striped bar), and scc-U8 (white bar) treated with cetuximab-IRDye700DX using a 24 h DLI and illuminated with different fluence rates to a fluence of 15 J·cm^−2^ for OSC-19-luc2-cGFP and scc-U2 and 7 J·cm^−2^ for scc-U8. † statistically significant from all other fluence rates investigated with the same cell line with *p* < 0.05, ‡ statistically significant from all other fluence rates investigated with the same cell line with *p* < 0.01.

The effect of fluence rate on the cell survival was investigated on the three head and neck cell lines OSC-19-luc2-cGFP, scc-U2 and scc-U8 with an EGFR expression relative to the expression in the OSC-19-luc2-cGFP (set to 100%) of 66% in scc-U8 and 70% in scc-U2 as previously determined [23]. Illumination with 150 mW·cm^−2^ after 24 h incubation with cetuximab-IRDye700DX resulted in 53 ± 6%, 69 ± 11%, and 83 ± 10% cell survival in the OSC-19-luc2-cGFP, scc-U2 and scc-U8 cell lines respectively (Figure 1). The total fluence delivered was 15 J·cm^−2^, 15 J·cm^−2^, and 7 J·cm^−2^ respectively to obtain a similar cell survival for the different cell lines [23]. Reducing fluence rate from 150 to 100 mW·cm^−2^, without changing the total fluence, does not decrease cell survival statistically significant for all three cell lines. Only when fluence rate was reduced to 75 mW·cm^−2^ cell survival was statistically significantly decreased with *p* < 0.05 compared to other fluence rate used for scc-U2 and OSC-19-luc2-cGFP. Further reduction of fluence rate to 20 mW·cm^−2^ resulted in a significantly decreased survival of 10 ± 2% for OSC-19-luc2-cGFP (*p* < 0.05), 12 ± 5% for scc-U2 (*p* < 0.01), and 22 ± 1% for scc-U8 (*p* < 0.01) compared to all other fluence rates used suggesting that oxygen availability can be an issue.

### 2.2. Formation of Singlet Oxygen

#### 2.2.1. Quenching Singlet Oxygen

**Figure 2:**
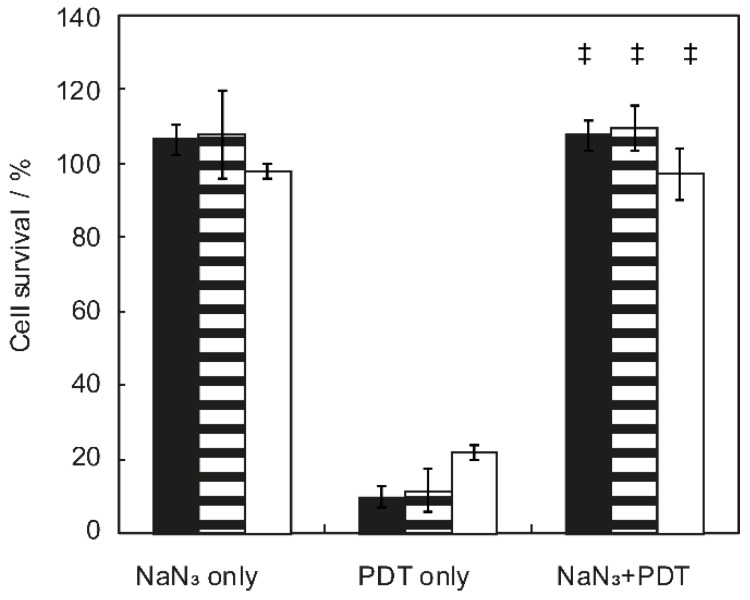
Cell survival of OSC-19-luc2-cGFP (black bar), scc-U2 (dashed bar), and scc-U8 (white bar) cells treated with either NaN_3_ only, cetuximab-IRDye700DX mediated photodynamic therapy (PDT) or the combination using a 24 h DLI and illuminated with 20 mW·cm^−2^ to a fluence of 15 J·cm^−2^ for OSC-19-luc2-cGFP and scc-U2 and 7 J·cm^−2^ for scc-U8. ‡ statistically significant from PDT only with *p* < 0.001.

PDT treatment in the presence of the singlet oxygen quencher NaN_3_ significantly diminished the effect of PDT with *p* < 0.001 in all cases (Figure 2). No significant difference in cell survival could be observed between NaN_3_ only and NaN_3_ + PDT treated cells for all three cell lines investigated suggesting that cell death after PDT is singlet oxygen dependent.

#### 2.2.2. Detection of Singlet Oxygen

To investigate the formation of singlet oxygen during in-vitro PDT, SOSG-EP fluorescence was imaged using confocal fluorescence microscopy. Control experiments, imaging scc-U8 cells incubated with SOSG for 2 h, confirmed that imaging with 488 nm could result in self-sensitized singlet oxygen as shown by the increase of SOSG-EP fluorescence in absence of cetuximab-IRDye700DX (Figure S1). Imaging with very low power 488 nm light did not result in an increase in SOSG-EP fluorescence. Additionally, imaging with 633 nm light, to excite cetuximab-IRDye700DX or 690 nm light to perform PDT, did not result in any increase of SOSG-EP. Similar results were obtained for scc-U2 cells (data not shown). Based on these experiments we chose to image SOSG-EP with very low intensity 488 nm light and collect a maximum of 10 images during illumination.

**Figure 3:**
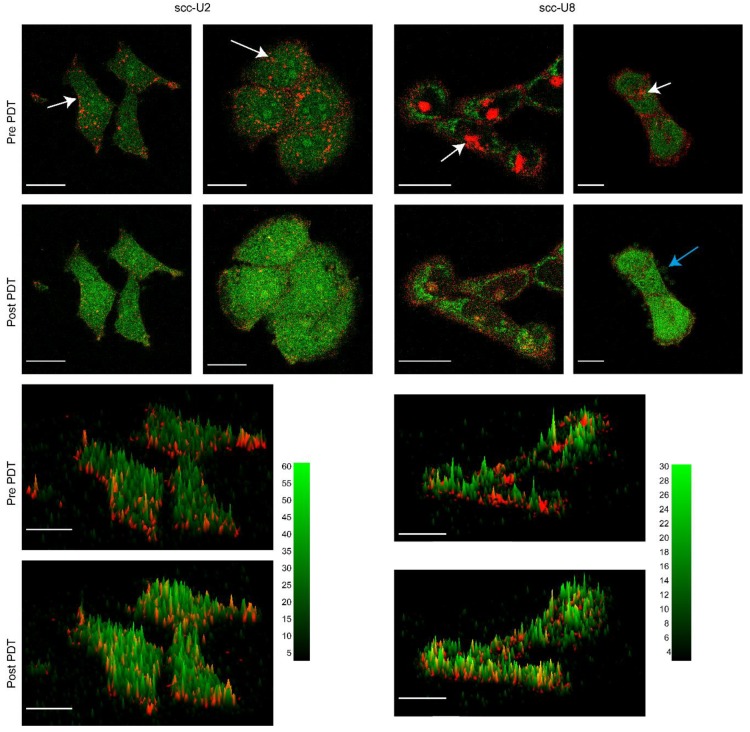
Examples of 2D images and 3D surface plots from images collected pre and post PDT of scc-U2 and scc-U8 cells incubated with cetuximab-IRDye700DX for 24 h and singlet oxygen sensor green (SOSG) for 2 h. White arrows point to some of the endo/lysosomes showing cetuximab-IRDye700DX fluorescence. Blue arrow point to blebs formed in response to the PDT treatment. Bar is 20 µm. Height of the peaks correspond to the fluorescence intensities of SOSG (green).

Incubation of cetuximab-IRDye700DX for 24 h resulted in fluorescence at the membrane and in the endo/lysosomes in both cell lines as shown in Figure 3 and in agreement with what is shown previously [23]. Confocal imaging after 2 h of SOSG incubation and using the established illumination parameters showed auto fluorescence and low levels of SOSG-EP fluorescence. After illumination, cetuximab-IRDye700DX fluorescence decreased and an increase in SOSG-EP fluorescence was observed. This increase in SOSG-EP fluorescence was not limited to the location of cetuximab-IRDye700DX but observed throughout the cytosol of the cells (Figure 3).

**Figure 4:**
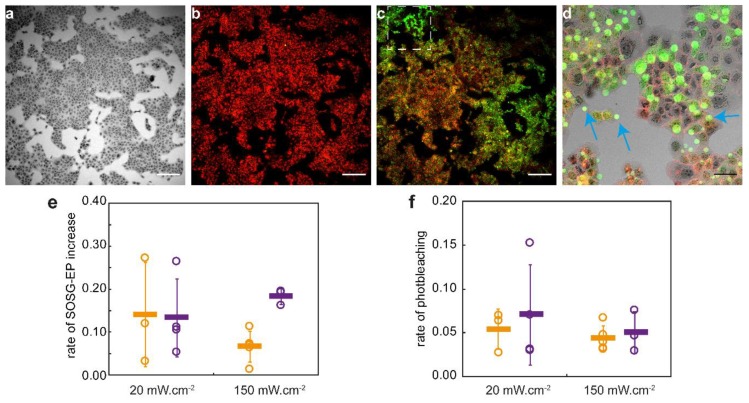
Example of a set of fluorescence images recorded of scc-U8 cells incubated with cetuximab-IRDye700DX for 24 h and SOSG for 2 h, illuminated with 20 mW·cm^−2^ to a fluence of 7 J·cm^−2^. (**a**) transmission image of cells pre illumination. (**b**) Cetuximab-IRDye700DX fluorescence (red) and SOSG fluorescence (green) at the start of illumination. (**c**) Cetuximab-IRDye700DX fluorescence (red) and SOSG fluorescence (green) at the end of illumination. (**d**) Zoomed in image of region shown in image c. White bar is 200 µm and black bar is 50 µm. Blue arrows point to examples of blebs formed in response to the PDT treatment. (**e**) The rate of SOSG-EP fluorescence increase in counts per J·cm^−2^ during illumination with either 20 or 150 mW·cm^−2^ in scc-U2 (orange) and scc-U8 (violet) cells. Open circles express the weighted mean for a set of images as shown in a-c and the solid bar expresses the weighted mean over 3–5 sets of images. (**f**) The rate of cetuximab-IRDye700DX photobleaching in counts per J·cm^−2^ during illumination with either 20 or 150 mW·cm^−2^ in scc-U2 (orange) and scc-U8 (violet) cells. Open circles express the weighted mean for a set of images as shown in a-c and the solid bar expresses the weighted mean over 3–5 sets of images.

Low magnification imaging during illumination gave the opportunity to investigate multiple cells. Figure 4 shows a gradual increase in SOSG-EP fluorescence in both scc cell lines. At the start of illumination almost no SOSG fluorescence was detectable (Figure 4b). During PDT SOSG fluorescence increased which varied from cell-to-cell (Figure 4c,d). No statistically significant difference was observed in mean SOSG-EP fluorescence increase and rate of increase after illumination with 20 or 150 mW·cm^−2^ (Figure 4e). We also observed no significant difference in extent and rate of cetuximab-IRDye700DX fluorescence photobleaching after illumination with 20 or 150 mW·cm^−2^ (Figure 4f). This is in agreement with the SOSG results and suggest that, given the illumination conditions of this part of the study, the rate of singlet oxygen formation is similar for the different fluence rates investigated.

### 2.3. Single Fiber Reflectance and Fluorescence Spectroscopy on Tumor Surface In-Vivo

#### 2.3.1. Reflectance Spectroscopy

**Figure 5:**
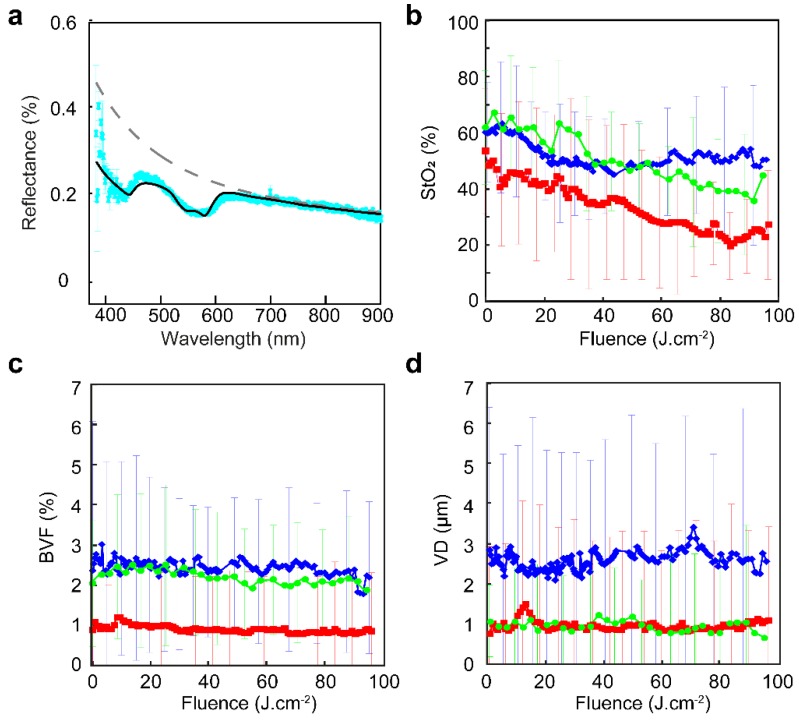
Reflectance measurements during PDT using different fluence rates. (**a**) Example of a collected reflectance spectrum recorded pre illumination (cyan), fitted spectrum (black solid line), and scattering background (dashed line) to determine StO_2_, BVF, and VD. (**b**) Weighted mean StO_2_ determined during PDT at 20 mW·cm^−2^ (blue diamonds), 50 mW·cm^−2^ (red squares), and 150 mW·cm^−2^ (green circles). (**c**) Weighted mean blood volume fraction (BVF) determined during PDT at 20 mW·cm^−2^ (blue diamonds), 50 mW·cm^−2^ (red squares), and 150 mW·cm^−2^ (green circles). (**d**) Weighted mean vessel diameter (VD) determined during PDT at 20 mW·cm^−2^ (blue diamonds), 50 mW·cm^−2^ (red squares), and 150 mW·cm^−2^ (green circles). No significant differences in vessel density and blood volume fraction were observed between tumors in each group.

Single fiber reflectance spectroscopy was used to monitor the response to PDT in the surface of OSC-19-luc2-cGFP tumors located subcutaneously on the flank of BALB/c nu/nu mice. An example of a collected reflectance spectrum, the fitted spectrum and the scattering background model in absence of absorption is shown in Figure 5a. Blood oxygen saturation (StO_2_) in this example was 14.9 ± 25.6%, blood volume fraction (BVF) was 4.2 ± 0.6% and mean vessel diameter (VD) was 5.6 ± 0.7 µm.

Figure 5b–d shows the weighted mean of four animals for blood oxygen saturation, blood volume fraction and mean vessel diameter during illumination with either 20, 50, or 150 mW·cm^−2^. No significant difference between the groups could be determined. During PDT no systematically significant changes occurred, except that blood oxygen saturation gradually decreased. We found no correlation between initial individual values for these vascular parameters and corresponding subsequent tumor growth delay.

#### 2.3.2. Fluorescence Spectroscopy

**Figure 6:**
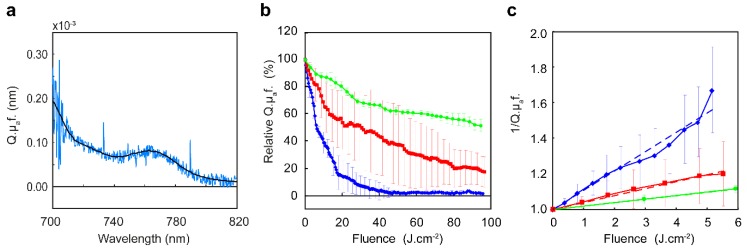
Fluorescence measurements during PDT using different fluence rates. (**a**) Example of a collected fluorescence spectrum recorded during illumination (azure blue) and the IRDye700DX basis spectrum (black). (**b**) Weighted mean intrinsic fluorescence intensity determined during PDT at 20 mW·cm^−2^ (blue diamonds), 50 mW·cm^−2^ (red squares), and 150 mW·cm^−2^ (green circles). (**c**) Weighted mean of reciprocal of normalized fluorescence during the first 5 J·cm^−2^ delivered at 20 mW·cm^−2^ (blue diamonds), 50 mW·cm^−2^ (red squares), and 150 mW·cm^−2^ (green circles) and their corresponding linear regression line.

Single fiber fluorescence spectroscopy was used in combination with reflectance spectroscopy to determine the intrinsic IRDye700DX fluorescence intensity in tumor. Figure 6a shows an example of a fluorescence spectrum recorded during illumination. Fluorescence kinetics during illumination is shown in Figure 6b,c. The total amount of photobleaching, reached at the end of illumination, and the initial rate of photobleaching in the first 10 J·cm^−2^ was significantly different between the three fluence rates used. Illumination with 20 mW·cm^−2^ to 100 J·cm^−2^ resulted in photobleaching to 1.5 ± 0.8 % of the initial fluorescence intensity, however already after approximately 30 J·cm^−2^ the weighted mean fluorescence intensity was not significantly different from zero. Illumination with 50 mW·cm^−2^ to 100 J·cm^−2^ resulted in photobleaching to 17.7 ± 11.2% which was significantly more compared to 20 mW·cm^−2^ (*p* < 0.05). Illumination with 150 mW·cm^−2^ to 100 J·cm^−2^ resulted in photobleaching to 51.6 ± 4.7% of the initial fluorescence which was significantly more than the two other treatments with *p* < 0.01. The presence of fluorescence at the end of illumination with 50 and 150 mW·cm^−2^ suggests that these treatments were less effective.

The initial photobleaching of cetuximab-IRDye700DX followed a second order decay as illustrated by the linearity of the reciprocal of the normalized fluorescence, as a function of light fluence (Figure 6c). Illumination with 20 mW·cm^−2^ led to a significantly faster photobleaching rate with respect to the fluence delivered in comparison to 50 and 150 mW·cm^−2^ with *p* < 0.05. Illumination with 50 mW·cm^−2^ led to a faster photobleaching rate with respect to the fluence delivered compared to 150 mW·cm^−2^ but this was not significant for the first 5 J·cm^−2^.

### 2.4. Tumor Response to PDT

**Figure 7:**
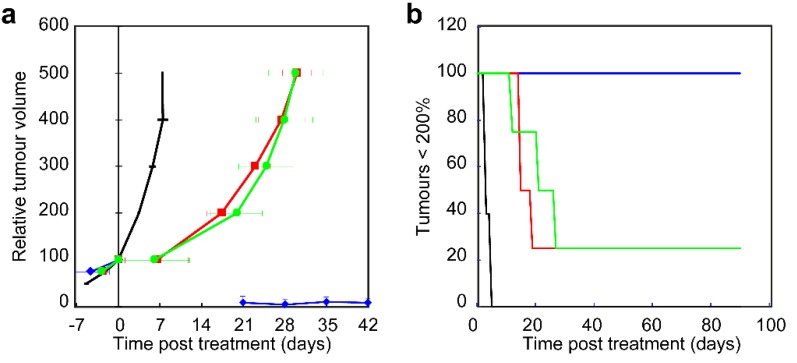
Effect of PDT using different fluence rates on the growth of OSC-19-luc2-cGFP tumor. (**a**) Relative tumor volume in time after treatment with control (black), 20 mW·cm^−2^ (blue diamonds), 50 mW·cm^−2^ (red squares), and 150 mW·cm^−2^ (green circles). (**b**) Kaplan–Meier plot of the percentage of tumors that didn’t grow to more than 200% of the treatment volume after treatment with control (black), 20 mW·cm^−2^ (blue), 50 mW·cm^−2^ (red), and 150 mW·cm^−2^ (green).

The mean tumor treatment volume for all treated animals before light exposure was 58.9 ± 11.4 mm^3^ (n = 17, range 30.4–77.2 mm^3^) and not significantly different between the four groups (ANOVA, *p* = 0.07). Control animals, receiving no drug and no light, showed a mean tumor volume doubling time of 3.4 ± 0.3% days (Figure 7a).

In the first few days after PDT treatment it was difficult to measure volumes of treated tumors due to edema in the illuminated area. Illumination with a fluence rate of 20 mW·cm^−2^ resulted in crust formation of skin and hemorrhage underneath skin. This was not observed in animals treated with 150 mW·cm^−2^.

In the 20 mW·cm^−2^ group, two out of four animals showed no tumor after the edema resolved up to 90 days post treatment and were considered a cure. One animal showed a small residual mass up to 50 days post treatment after which this was not palpable for the rest of the observation period, also considered cured. And one animal showed a residual tumor that decreased in volume up to day 50 post treatment and remained stable around 26% of the initial volume up to day 90 post treatment. Immuno-histological and H&E staining of the formalin fixed paraffin-embedded (FFPE) tissue showed negative staining for pan-Keratin and CD45 suggesting non-vital tissue (Figure S2). Note that the lack of regrowth of the tumor results in a relative low mean tumor volume in the growth curve of Figure 7a. This data is better visualized using a Kaplan Meijer plot of the percentage of tumors that didn’t grow larger than 200% of the treatment volume (Figure 7b).

In the 50 mW·cm^−2^ group 1 out of 4 animal showed residual tumor that remained stable between 100% and 150% of the initial tumor volume up to 90 days post treatment. H&E staining of the FFPE tissue showed cell rich fibrotic tissue with areas of necrosis and thickened blood vessel walls. Immuno-histological analysis showed negative staining for pan-Keratin and CD45 suggesting this is not vital tissue (Figure S2). The other three animals showed tumor regrowth to more than 200% of their initial treatment volumes within 16.2 ± 2.2 days.

In the 150 mW·cm^−2^ group 1 out of 4 animals showed no regrowth of tumor from day 13 to 90 days post treatment and was considered cured. The other three tumors showed regrowth of tumor to more than 200% of their initial treatment volumes within 19.9 ± 7.4 days, not significantly different from 50 mW·cm^−2^.

## 3. Discussion

We have demonstrated that tumor growth delay and in-vitro cell survival show a clear dependence between fluence rate and efficacy during EGFR targeted PDT. Significantly less cell survival is observed using a fluence rate of 20 mW·cm^−2^ compared to 150 mW·cm^−2^ in-vitro and significant less tumors were cured after illumination with 150 mW·cm^−2^ or 50 mW·cm^−2^ compared to 20 mW·cm^−2^ in-vivo. It is well known that fluence rate is an important factor for therapeutic outcome of non-targeted PDT [3,4,10,12]. Illumination with a high fluence rate has shown to result in less tissue damage during 5-aminolevulinic acid based protoporphyrin IX PDT [3]. Similar results were reported on tumor growth after Photofrin [28] and 2-[1-hexyloxyethyl]-2-devinyl pyropheophorbide-a mediated PDT [29]. Foster et al. already described in 1991 that oxygen consumption for singlet oxygen formation during a high fluence rate illumination exceeds the rate at which oxygen can be resupplied from the vasculature [30]. Schouwink et al. showed for mTHPC that the median *p*O_2_ significantly dropped in RIF1 tumors during illumination with 100 mW·cm^−2^ [31]. Coutier et al. reported for mTHPC that illumination with a fluence rate of 30 and 5 mW·cm^−2^ did not change the intratumor *p*O_2_ and resulted in a significant longer tumor growth delay compared to higher fluence rate illuminations [32]. The rate and extent of photobleaching can be used for implicit dosimetry [3,10,11,12]. In the present study we observed a slower rate of photobleaching for high fluence rate illuminations of 50 and 150 mW·cm^−2^ compared illumination with 20 mW·cm^−2^. We also observed less photobleaching for high fluence rate illuminations. Here the effective PDT dose delivered during a high fluence rate illumination is lower than during a low fluence rate. The differences in cell survival, tumor response and cures in the present study are clearly the result of a difference in effective PDT dose delivered, due to the choice of fluence rate. Targeted photosensitizers are potentially sensitive to oxygen depletion since they are designed to deliver the photosensitizer to tumor cells at high concentrations using a targeting molecule that is aimed at overexpressed receptors and usually conjugated with more than 1 photosensitizer molecule. A recent study reported that the degree of conjugation of IRDye700DX to cetuximab was approximately 3 [20]. We expect that the clinical formulation of cetuximab-IRDye700DX will have a similar degree of conjugation. During illumination this results in a high demand for oxygen in those targeted tumor cells that are far from the oxygen supply. High fluence rate illumination might therefore be particularly disadvantageous for targeted PDT using cetuximab-IRDye700DX.

Although illumination with a low fluence rate of 20 mW·cm^−2^ showed significant better responses, the duration of the treatment might be a problem clinically. Fluorescence data showed complete photobleaching long before the end of illumination with 20 mW·cm^−2^. From studies investigating 5-aminolevulinic acid mediated PDT it is known that continuing illumination after complete PpIX photobleaching does not lead to an increase in PDT efficacy [33]. We have shown previously, in ALA mediated PDT, that the normalized extent of photobleaching can be used as a predictor of PDT response [3,33,34]. It is likely that this also applies for IRDye700DX and it would be interesting to investigate this, as for clinical implementation shortening the low fluence rate illumination without loss of efficacy would be beneficial.

Cetuximab-IRDye700DX localizes at the membrane and in the endo-lysosomes of the cell supporting our previous results [23]. In general, photosensitizers accumulated in the endo-lysosomes become aggregated. Upon illumination the endo-lysosomes disrupt, content is released into the cytosol and aggregated photosensitizers become de-aggregated. While the aggregated state of a photosensitizer, in general, has a low quantum yield de-aggregation of a photosensitizer would lead to an increase in fluorescence and photoactivity [35,36]. Hypothetically, fluorescence intensity would increase shortly after the start of illumination and the higher efficacy of low fluence rate illuminations would be due to a longer illumination time. Our current results, however, show a continues decrease in fluorescence intensity during illumination suggesting that this effect is not occurring in EGFR-targeted PDT.

We found no correlation between treatment outcome and vascular parameters monitored during PDT. Blood oxygen saturation, blood volume fraction, and mean vessel diameter did not show any clear changes during illumination at different fluence rates. Cetuximab is a chimeric (mouse/human) monoclonal antibody and therefore not expected to bind to mouse EGFR. Vascular responses due to direct damage of endothelial cells was therefore not expected and the current results show that also indirect vascular responses are not occurring during PDT. We observed hemorrhage at 1 or 2 days post treatment but this was not related this to an increase in blood volume fraction during PDT. This suggests that the majority of vascular responses occur after PDT. This observation is supported by a previous study in which we showed vascular leakage and vasoconstriction after PDT using cetuximab-IRDye700DX [24]. Our photobleaching results suggest oxygen depletion but vascular monitoring shows no vasoconstriction. This confirms that the rate of oxygen consumption for the singlet oxygen generation indeed exceeds the rate at which oxygen can be resupplied from the vasculature during high fluence rate illuminations. The limiting factor most likely is diffusion of oxygen from vessels to cells.

Reports in literature on the role of oxygen availability and the formation of singlet oxygen in the response to antibody targeted photosensitizers in-vitro are inconclusive [18,19,22]. Both Mitsunaga et al. [18] and Shirasu et al. [19] show only partial reduction in effectiveness of antibody-PS mediated PDT in the presence of sodium-azide, a known singlet oxygen quencher. Whereas Kishimoto et al. [22] found complete loss of effectiveness in similar experiments investigating a different antibody-PS conjugate. While we previously reported that scc-U2 and ssc-U8 have different sensitivities to targeted PDT, in the present study we demonstrate that both cell lines show increasing cell death for lower fluence rates. Furthermore we were able to diminish the effect of treatment completely when illuminated in the presence of NaN_3_ supporting our hypothesis that singlet oxygen is the source of damage in targeted PDT. Mitsunaga et al. [18] already showed that the capacity of NaN_3_ to quench singlet oxygen was dose dependent suggesting that the NaN_3_ dose used, and probably also the NaN_3_ incubation times applied, might play a role in the reported inconsistencies.

Involvement of singlet oxygen in the response to targeted PDT is further confirmed, in the present study, by the observed increase in fluorescence of singlet oxygen sensor green during illumination. Gollmer et al. demonstrated the drawbacks utilizing SOSG as an accurate and reproducible probe for intracellular singlet oxygen [37]. For example, binding of SOSG to a protein in medium could adversely influence a number of processes by which SOSG could enter the cell. For this reason, in the present study, cells were incubated with SOSG in saline. Intracellularly SOSG will bind to proteins but this will not prevent SOSG to react with singlet oxygen to form SOSG-EP and fluoresce, as shown by Gollmer et al. [37]. We performed controls to define the imaging conditions to make sure that SOSG-EP fluorescence increase was due to singlet oxygen formed by illuminated cetuximab-IRDye700DX. Our high magnification imaging of single cells, or small clusters of cells, showed SOSG-EP fluorescence increase throughout the whole cell and not just localized to the location of cetuximab-IRDye700DX. Gollmer et al. found that it was difficult to obtain systematic and reproducible results upon irradiation of SOSG-containing cells, some cells showed clearly increases in fluorescence intensity of SOSG-EP, whereas other cells did not [37]. We observed this also in the current study. For this reason, we continued our study imaging a large proportion of a 96 well, containing numerous cells to determine the SOSG-EP fluorescence increase. The increase in SOSG-EP fluorescence during PDT using different fluence rates was however not significantly different, suggesting that oxygen availability was not limited during high fluence rate illumination. This is in disagreement with the results on cell survival where we did see differences between illumination with high or low fluence rates. This discrepancy between results is probably related to differences in experimental circumstances. The cell survival results were obtained with 100 μL medium in each well and the 96-wells plate placed on a shaker to facilitate oxygen diffusion as much as possible. The SOSG-EP fluorescence measurements were performed on static microscopic stage and with only 50 μL of saline in each well. The diffusion of oxygen from air through fluid to cells is highly dependent on the level of fluid on top of the cells [38]. Furthermore the oxygen tension in water is higher than in medium [39]. While we have no data on cell death after SOSG-EP fluorescence measurements it might be that the difference in experimental conditions account for the difference in fluence rate effects observed.

Two of the treated tumors, one treated with 20 mW·cm^−2^ and another treated with 50 mW·cm^−2^ were still palpable after 90 days but did not regrow. H&E and immune-histological staining of these tissues showed cell rich fibrotic tissue and no sign of inflammation or vital tumor tissue. These two tumors were not considered as unsuccessful treated since it did not grow back to more than 200% but also not considered as cured. It is unclear why the treated tissue remained present in the mouse and was not cleared. Tissue remaining present at the treated tumor or lymph node sites is occasionally observed in patients treated for head and neck cancer. Establishing that the treatment killed all viable tumor cells is known to be difficult even on excised tissues and possess a clinical issue.

The data presented here show that a low fluence rate used for EGFR-targeted PDT improves the treatment outcome in an immune compromised nude mouse model. It is known that the immune response contributes to the PDT response and that this is higher after low fluence rate compared to high fluence rate illumination [4,29,40]. Although this needs to be confirmed in a model with a functional immune system, it is likely that this is also true for targeted PDT. From a clinical perspective the use of low fluence rate illumination may therefore even be more beneficial.

In summary, we show that the source of damage in antibody EGFR targeted PDT is the generation of singlet oxygen. The effectiveness of targeted PDT is dependent on the fluence rate used. Clinical PDT may benefit from a low fluence rate illumination and might be dosed on the photobleaching of the photosensitizer.

## 4. Materials and Methods 

### 4.1. Cell Lines and Culture

Three human head and neck (oral cavity) squamous cell carcinoma cell lines, scc-U2, scc-U8 (University of Michigan), and OSC-19-luc2-cGFP [41] (University of Leiden) were used in this study. The scc-U2, and scc-U8 cell lines were cultured in Dulbecco’s Modified Eagle Medium (DMEM, Gibco, ThermoFisher scientific, Waltham, MA, USA) supplemented with 10% fetal calf serum (FCS, Lonza, Basel, Switzerland), 100 U/mL penicillin, 100 mg/mL streptomycin, and 2 mM glutamine (Gibco, ThermoFisher scientific, Waltham, MA, USA) at 37 °C in a humidified 5% CO_2_ atmosphere. The OSC-19-luc2-cGFP cell line was cultured in advanced DMEM (Gibco, ThermoFisher scientific, Waltham, MA, USA) containing 4.5 g/L D-glucose, 110 mg/L sodium pyruvate, 580 mg/L glutamine supplemented with 10% FCS (Lonza, Basel, Switzerland), 1400 IU/mL penicillin, 100 mg/mL streptomycin, 1× Minimal Essential Medium (MEM) non-essential amino acids solution, and 1× MEM vitamin solution (Gibco, ThermoFisher scientific, Waltham, MA, USA) at 37 °C in a humidified 5% CO_2_ atmosphere. The passages of 10–40 of the cell lines were used in this study.

### 4.2. Targeted Photosensitizer

Cetuximab-IRDye700DX was provided by Aspyrian Therapeutics, Inc., San Diego, CA, USA.

### 4.3. In-Vitro PDT Illumination

Each cell line was seeded with 15,000 cells in each well of a 96-well plate. After 24 h incubation for attachment, the cells were incubated in the dark with 40 μg/mL cetuximab-IRDye700DX for 24 h at 37 °C. Cells were washed and a volume of 100 μL medium was added to the cells. Illumination was performed with a 690 nm laser (Modulight ML7700, Tampere, Finland) using a shaker with a speed of 700 RPM to facilitate oxygen diffusion as much as possible. The fluence rate was determined at the surface of the 96 well plate to account of scattering and reflection of the incident irradiance using an isotropic detector (Medlight SA, Ecublens, Switzerland) connected to an optical power meter (National Instruments NI PXI-1045 and PX 2000-306, Woerden, The Netherlands). Cells were illuminated with a fluence rate of 150, 125, 100, 72, or 25 mW·cm^−2^. The total fluence delivered was different for the different cell lines to compensate for the difference in sensitivity to PDT treatment. Scc-U8 cells were illuminated to a fluence of 7 J·cm^−2^ and scc-U2 and OSC-19-luc2-cGFP cells to a fluence of 15 J·cm^−2^ [23]. Cell survival was determined 24 h after PDT using the cell proliferation MTS assay. Since previous reports from us and others have shown very little photodynamic effect on cells using IRDye700DX alone, we chose not to include it as a control [18,42].

### 4.4. Cell Survival

Cell proliferation was assessed with a standard MTS kit (CellTiter 961 AQueous One Solution Reagent (Promega Corp., Madison, WI, USA) according to the manufacturer’s recommendations using a 96-well plate reader (Molecular Devices, Sunnyvale, CA, USA). The formazan product that is detected by the 492 nm absorbance was not influenced by the IRDye700DX.

### 4.5. Formation of Reactive Oxygen Species

The formation of singlet oxygen formation was investigated using two different experiments. In the first experiment PDT was performed in the presence of the singlet oxygen quencher sodium azide. In the second experiment the formation of singlet oxygen was visualized using confocal imaging during PDT in the presence of singlet oxygen sensor green (SOSG, Molecular Probes, Oregon, OR, USA).

#### 4.5.1. Quenching Singlet Oxygen

Each cell line was seeded with 15,000 cells in each well of a 96-well plate. After 24 h incubation for attachment, the cells were incubated in the dark with 40 μg/mL cetuximab-IRDye700DX for a total of 24 h at 37 °C. After 20 h incubation with cetuximab-IRDye700DX, sodium azide (NaN_3_) at 50 mM (Sigma-Aldrich, Zwijndrecht, NL, USA) was added for the remaining 4 h. Cells were then washed at a volume of 100 μL medium before illumination. PDT and cell survival measurements were performed in the same manner as described previously (in-vitro PDT illumination).

#### 4.5.2. Detection of Reactive Oxygen Species

Scc-U2 and scc-U8 cell lines were used to investigate the formation of singlet oxygen using SOSG during confocal imaging. This assay could not be performed on OSC-19-luc2-cGFP due to the GFP expression of the cell line which conflicts with the fluorescence detection of SOSG endoperoxide (SOSG-EP), the product of the reaction between SOSG and singlet oxygen.

The scc-U2 and scc-U8 cell lines were seeded with 8000 cells in a well of a 96-well plate (one well per plate to avoid cross illumination). After 24 h incubation for attachment, the cells were incubated in the dark with 40 µg/mL cetuximab-IRDye700DX at 37 °C. After 24 h cells were washed 3 times with saline and incubated with singlet oxygen sensor green (10 μM in saline) for 2 h at 37 °C. After washing 3 times with saline the cells were supplied with 50 μL saline and placed on the heated stage of a Leica SP5 Microscope equipped with a 10× Plan-Neofluar objective. It is well known that SOSG and SOSG-EP can themselves both act as photosensitizers [37,43], therefore the imaging parameters (488 nm excitation light intensity), pixel dwell time and number of images collected were carefully defined in control experiments on cells incubated with only SOSG for 2 h.

The conjugate fluorescence was recorded before and after illumination using 633 nm excitation and 650–800 nm HyD detection. The SOSG-EP fluorescence images were recorded before, during and after illumination using 690 nm light delivered at either 150 mW·cm^−2^ or 20 mW·cm^−2^ to a fluence of 15 J·cm^−2^. Cells illuminated with 150 mW·cm^−2^ were imaged 3 times; immediately before, halfway (50 s) and immediately after illumination. Cells illuminated with 20 mW·cm^−2^ were imaged 9 times; before, every 100 s during illumination and after illumination. ImageJ 1.48v was used to analyze the images recorded. The pre-PDT fluorescence image of cetuximab-IRDye700DX was used to create regions of interest (ROI) around the individual or small clustered cells by the thresholding and analyze particles feature. This was used to determine the fluorescence intensity in both the red and the green channel in the images recorded. The SOSG-EP fluorescence and cetuximab-IRDye700DX fluorescence intensity was determined per cell or groups of cells and averaged (weighted mean) per image. Per cell line and fluence rate 3 to 5 sets of images were recorded and averaged (weighted mean). The rate of photobleaching was determined as the slope of the reciprocal of the normalized fluorescence using linear regression fitting. The rate of SOSG-EP fluorescence increase was determined as the slope of the fluorescence increase using linear regression fitting. Data of ROI’s with a max of 255 in the red channel, or a R^2^ lower than 0.90 in linear regression fitting were excluded from data analysis.

### 4.6. Solid Tumor Model

Female BALB/c nude mice (Charles River) aged 8–11 weeks were injected with 1 × 10^6^ OSC-19-luc2-cGFP cells subcutaneously in the flank. Volumes of the ellipsoid-shaped tumors were calculated from the 3 diameters that were measured using a caliper every 2 or 3 days after they became palpable. The formula used was V = (D1 × D2 × D3) × π/6. Only animals with tumors showing exponential growth were included in the study (80%). Experiments were started when the tumors reached a volume of approximately 40–70 mm^3^ and the treatment volume was defined 100%. After illumination tumors were again measured with a caliper every 2 or 3 days until 500% of the treatment volume or up to 90 days after treatment by HB and/or WP blinded to the treatment parameters. The points in time at which the regrown tumor reached a volume of respectively 100%, 200%, 300%, 400%, and 500% were linearly interpolated and averaged for each treatment group (n = 4–5 per group). The effectiveness of each treatment scheme was determined by comparison of the mean tumor volume doubling time, defined as the number of days the regrown tumor required to double its pre-treatment volume. A tumor is considered cured when it reduced in size until they were not palpable and remained so for 90 days after treatment. Tumor tissue that reduced in size but remains palpable and does not grow will be excised at 90 days post treatment for pan-Keratin and CD45 staining. The Netherlands National Committee for the protection of animals used for scientific purposes approved the protocol (AVD1010020151636). All applicable institutional and/or national guidelines for the care and use of animals were followed.

### 4.7. In-Vivo PDT Illuminiation

**Figure 8:**
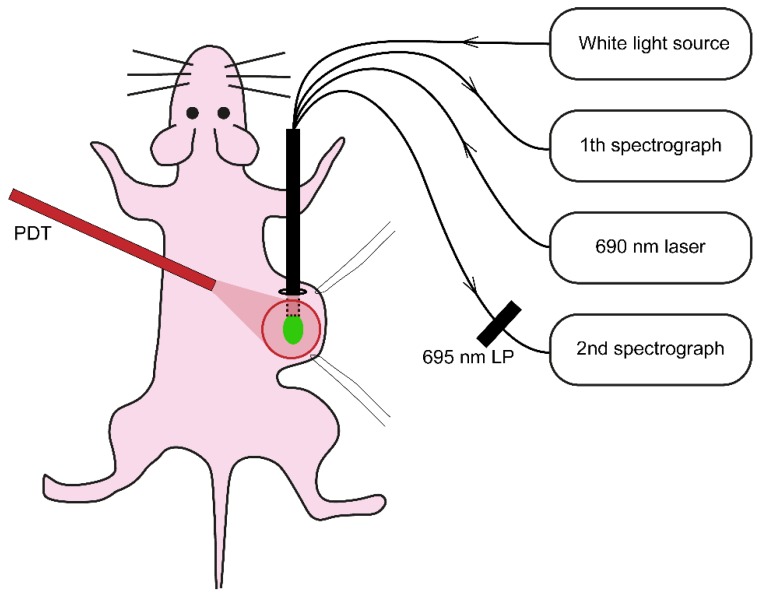
Schematic drawing of the illumination and single fiber spectroscopy set-up.

Illumination was performed under gas anesthesia 24 h after i.v. administration of cetuximab-IRDye700DX. Mice injected with physiological saline served as a light only control (n = 5). Just before illumination the skin overlying the tumor was loosely stretched using two sutures and the quadfurcated fiber, for collecting the reflectance and fluorescence spectra, was placed underneath the skin in touch with (but not pressing against) the tumor (Figure 8). Reflectance and fluorescence spectra were recorded before, during and until max 5 min after illumination without repositioning the fiber. A 690 nm laser (ML7700, Modulight Inc., Tampere, Finland) and a frontal light distributor (Medlight SA, Ecublens, Switzerland) were used to deliver a dose of 100 J·cm^−2^ at a of 20, 50, or 150 mW·cm^−2^ (n = 4 in each group).

### 4.8. Single Fiber Reflectance and Fluorescence Spectroscopy

The experimental setup used to collect reflectance and fluorescence spectra is based on what has been described in detail previously [44] and is shown in Figure 8. In short, the setup utilizes a single optical fiber connected to a quadfurcated optical fiber polished under an angle of 15° to minimize internal specular reflections from the fiber tip. White light reflectance spectra were obtained after excitation of white light from a halogen light source HL-2000-FHSA (Ocean Optics, Duiven, The Netherlands) via one arm and collection of the reflected light via a second arm connected to a spectrophotometer SD-2000 (Ocean Optics, Duiven, The Netherlands). A calibration procedure was performed to account for internal reflections, variability in lamp-specific output and in fiber-specific transmission properties [45]. Fluorescence spectra were obtained immediately after the reflection measurement using excitation light split from the 690 nm therapeutic laser (at a fluence rate that approximately matched the treatment fluence rate in each group) connected to the third arm of the quadfurcated fiber. The emitted fluorescence was collected through the fourth arm of the quadfurcated fiber, filtered through the 695 LP filter and connected to a second spectrograph (QE-6500, Ocean Optics, Duiven, The Netherlands).

### 4.9. Mathematical Analysis of Spectra

Single fiber reflectance spectra were analyzed using an analytical model to describe the wavelength-dependent optical properties to extract physiological and morphological information from the sampled tissue as previously described [13]. In short, attenuation due to absorption within the tissue is modeled using a modified Beer–Lambert law and is a function of both the tissue absorption coefficient (μ_a_) and the single fiber photon path length. The reflectance amplitude, as well as the single fiber photon path length, depend on the scattering properties of the tissue, with a dependence on the reduced scattering coefficient (μ′_s_) and on the angular distribution of scattering (phase function). The dependence of the single fiber reflectance signal on phase function parameter gamma results from the overlapping source–detector areas utilized in single fiber measurements, for which the diffusion approximation does not hold [46]. To fit the data, the fiber core diameter, numerical aperture of the fiber and the refractive index of the solid tumor under investigation was taken into account. The reduced scattering coefficient was fitted as a power-law function [45], and γ was assumed to be constant over the fitted wavelength range. Furthermore, instead of fitting γ as a free parameter, we kept *γ* fixed at 1.4, 1.6, and 1.8, which is an expected range for *γ* in biological tissues [47]. We have verified that this did not result in differences in the estimated absorption coefficient of more than 6% compared to other fitting approaches.

Since we have not previously performed measurements on the OSC-19-luc2-cGFP solid tumor, our initial model assumed that absorption was attributable to oxygenated (HbO_2_) and deoxygenated hemoglobin (Hb) confined within the local microvasculature and bilirubin. A Levenberg–Marquardt algorithm was used to estimate the parameter values for the microvascular hemoglobin oxygen saturation StO_2_, the blood volume fraction (BVF) and average blood vessel diameter (VD) by minimizing the chi-squared metric between measured reflectance data and model predictions. Confidence intervals on parameter estimates were calculated from the square root of the diagonal of the covariance matrix [48]. Parameter values were averaged over repeated measurements, weighted by the confidence interval of individual spectral fits, and reported with the associated weighted standard deviation.

The fluorescence spectra from all individual measurements were corrected for the absorption and scattering as described before [27]. Spectral deconvolution was used to divide the corrected fluorescence spectrum into autofluorescence and photosensitizer fluorescence. Subtraction of the autofluorescence spectrum then yields the intrinsic fluorescence spectrum. The resulted intrinsic photosensitizer fluorescence spectrum was related to the product of photosensitizer absorption coefficient µ_a_^f^ (mm^−1^) and quantum yield Qf (−) by integration over wavelength. A basis spectrum of IRdye700DX was constructed from 3 measurements on 3 different mice and used to fit the intrinsic fluorescence spectrum. From the fitted curve we were able to determine the intrinsic fluorescence intensity and the 95% confidence interval. The rate of photobleaching over the first 5 J·cm^−2^ was determined as the slope of the reciprocal of the normalized fluorescence using linear regression fitting.

### 4.10. Statistics

All results are presented as mean ± SD, except for data collected using single fiber spectroscopy. The fitted values for StO_2_, BVF, VD and Q.µ_a_^f^ were weighted by its confidence interval to result in a weighted mean ± SD. The significant difference was determined using the Student’s *t*-test/ANOVA/SNK and *p* < 0.05 was considered significant. For the tumor growth delay data also multivariate analysis and Chi quadrant analysis were used.

## 5. Conclusions

Our results show that the damage in targeted PDT is highly dependent on the fluence rate used. The effectiveness of targeted PDT is, like PDT, dependent on the generation of singlet oxygen and thus on the availability of intracellular oxygen.

## Supplementary Materials

The following are available online at https://www.mdpi.com/2072-6694/12/1/190/s1, Figure S1: Effect of imaging parameters and PDT illumination on the SOSG photosensitization of scc-U8 cell incubated with SOSG for 2 h, Figure S2: Microscopic images of sections stained with H&E and anti-pan Keratin or anti CD45 immunohistologically.
