# Supplementary material for: In-Vivo Optical Monitoring of the Efficacy of Epidermal Growth Factor Receptor Targeted Photodynamic Therapy: The Effect of Fluence Rate
Source: Cancers (Basel). 2020 Jan 13;12(1):190. doi: 10.3390/cancers12010190 (PMC7017190; doi:10.3390/cancers12010190)
Supplement: Supplementary file 1 [file cancers-12-00190-s001.pdf]

cancers

Supplementary Materials

In-Vivo Optical Monitoring of the Efficacy of Epidermal Growth Factor Receptor Targeted Photodynamic Therapy: The Effect of Fluence Rate

Henriette S. de Bruijn, Wei Peng, Timo L.M. ten Hagen, Kristian Berg, Jan L.N. Roodenburg, Go M. van Dam, Max J.H. Witjes, Dominic J. Robinson

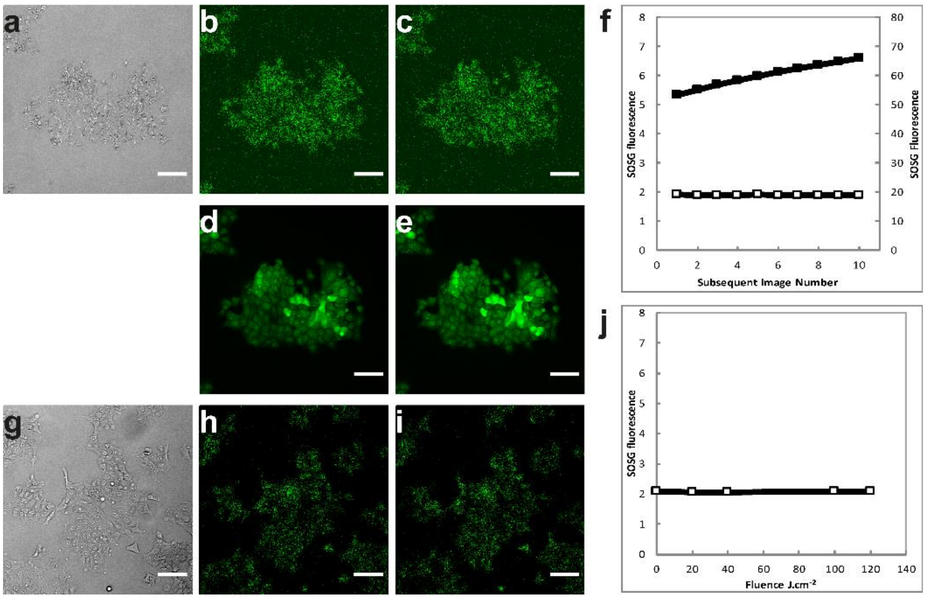

Figure S1. Effect of imaging parameters and PDT illumination on the SOSG photosensitization of scc-U8 cell incubated with SOSG for 2 h. (**a–e**) Set of images, collected using different illumination parameters, comprising (**a**) transmission image of cells, (**b**) first fluorescence image collected using 1% 488 nm light, (**c**) 10th fluorescence image collected using 1% 488 nm light, (**d**) first fluorescence image collected using 50% 488 nm light, (**e**) 10th fluorescence image collected using 50% 488 nm light. (**f**) SOSG-EP fluorescence intensity determined in the cells in consecutive images collected using 1% 488 nm light (open squares, left y-axis) and 50% 488 nm light (closed squares, right y-axis). (g–**i**) Different set of images, collected during PDT illumination with 690 nm light, using 1% 488 nm light and 633 nm light to collect cetuximab-IRDye700DX in future experiments, comprising (**g**) transmission image, (**h**) pre illumination and (**i**) post illumination. (**j**) SOSG-EP fluorescence intensity determined in the cells in consecutive images collected using 1% 488 nm light (open squares) during illumination with 690 nm light. Bar is 100 μm.

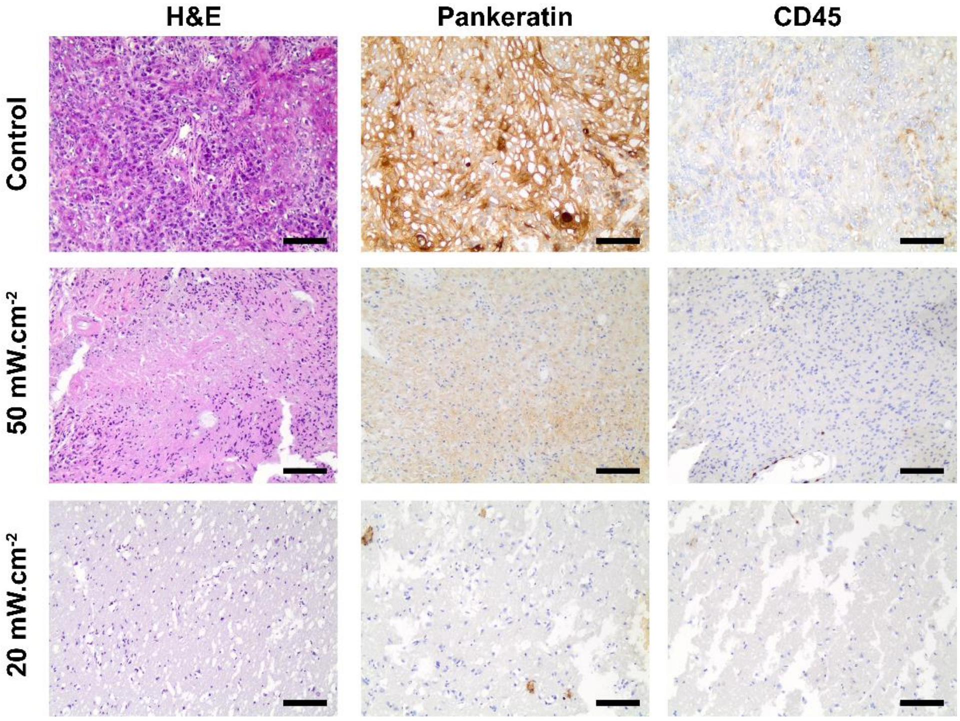

Figure S2. Microscopic images of sections stained with H&E and anti-pan Keratin or anti CD45 immunohistologically. Top panel depicts sections of control, untreated, tumor. Middle panel shows sections of residual tissue still palpable 90 days post treatment with 50 mW·cm^{-2}. And lower panel depicts the sections of residual tissue still palpable 90 days post treatment with 50 mW·cm^{-2}. Bar represents 100 μm.
